# Protocol for the economic evaluation of individualised (early) patient-directed rehabilitation versus standard rehabilitation after surgical repair of the rotator cuff of the shoulder (RaCeR 2)

**DOI:** 10.1136/bmjopen-2024-097469

**Published:** 2025-06-20

**Authors:** Andrea Manca, Shainur Premji, Vijay S Gc, T Chen, Bruno Mazuquin, Apostolos Fakis, Marcus Bateman, Maria Moffatt, Chris Littlewood

**Affiliations:** 1 Centre for Health Economics, University of York, York, England, UK; 2 Department of Clinical Sciences, Liverpool School of Tropical Medicine, Liverpool, UK; 3 Department of Health Professions, Manchester Metropolitan University, Manchester, UK; 4 Derby Clinical Trials Support Unit, Royal Derby Hospital, Derby, UK; 5 Derby Shoulder Unit, University Hospitals of Derby and Burton NHS Foundation Trust, Derby, UK; 6 Institute of Population Health, University of Liverpool Faculty of Health and Life Sciences, Liverpool, UK; 7 School of Health and Society, Salford, UK

**Keywords:** Health, REHABILITATION MEDICINE, Shoulder, HEALTH ECONOMICS

## Abstract

**Introduction:**

RaCeR 2 is a pragmatic multicentre, open-label, randomised controlled trial, with full economic evaluation. The primary aim is to assess whether individualised (early) patient-directed rehabilitation (EPDR) results in less shoulder pain and disability at 12 weeks postrandomisation following surgical repair of full-thickness tears of the rotator cuff of the shoulder compared with the current standard (delayed) rehabilitation. This paper provides the protocol for the RaCeR 2 health economic evaluation.

**Methods and analysis:**

The health economic analysis of RaCeR 2 is made up of three phases: (1) development of an initial state-transition model structure, (2) within-trial cost consequence analysis and (3) long-term model-based cost-effectiveness analysis (CEA) from the National Health Service and Personal Social Service perspective in England. Descriptive statistics (eg, mean, standard deviation, 95% confidence intervals and minimum and maximum values) will be reported for within-trial resource use, costs and health-related quality of life (HRQoL). Health state-specific costs and HRQoL will be estimated using regression model approaches and used to inform a state-transition simulation model designed to quantify the long-term costs and quality-adjusted life years (QALYs) experienced by patients over the model’s time horizon. Where appropriate, final CEA model results will be reported as cost per QALY gained for individualised EPDR versus standard (delayed) rehabilitation. Model assumptions and overall parameter uncertainty will be tested using probabilistic sensitivity analysis and scenario analyses. All regression analyses will be adjusted for baseline participant demographic and symptomatic characteristics.

**Ethics and dissemination:**

A favourable ethical review was granted by London-Stanmore Research Ethics Committee (23/LO/0195) on 13 April 2023. Findings will be disseminated in peer-reviewed journals, at scientific conferences, and via the study website.

**Trial registration number:**

ISRCTN11499185

**STRENGTHS AND LIMITATIONS OF THIS STUDY**

- This protocol provides a detailed roadmap for an economic evaluation study that uses prospectively collected data from the RaCeR 2 randomised controlled trial.
- We outline the study objectives, methods and statistical considerations, serving as a guide for the research team and facilitating peer review and reproducibility assessment.
- We will analyse individual patient-level resource use, costs and resulting health outcomes observed during study follow-up.
- We will work collaboratively with patient and public involvement to classify patients into symptom states for our de novo Markov state transition model.
- To characterise remaining uncertainties in overall cost-effectiveness, we will use the value of information analysis to assess the risk or consequences of implementing individualised (early) patient-directed rehabilitation within National Health Service England.

##  Introduction

Shoulder pain is one of the most common reasons for general practitioner (GP) consultations in the United Kingdom (UK) and is associated with age, smoking, occupation and socioeconomic status.^1^,^3^ With a prevalence of approximately 87 per 100 000 person-years, it is more common in women than men^2^ and can result in significant disability, disturbed sleep and overall quality of life losses.^3 4^ Shoulder pain can be caused by a range of problems including partial or full-thickness rotator cuff tears.^1 5^ Commonly used strategies to support people with rotator cuff disorders include activity modification, corticosteroid injections, exercise therapy and/or surgery.^6^,^8^

While surgical techniques have progressed, the optimal approach to rehabilitation following surgery to repair the torn rotator cuff remains unknown.^5 9^ Current standard rehabilitation in the UK National Health Service (NHS) typically includes sling immobilisation for approximately 4 weeks postsurgery.^10^

Following an initial pilot randomised controlled trial (RCT) with feasibility objectives,^5^ RaCeR 2 was designed as a pragmatic multicentre, open-label, RCT using a parallel group design with a 1:1 allocation ratio, full economic evaluation and integrated Quintet Recruitment Intervention.^11^ The aim of RaCeR 2 is to assess whether individualised (early) patient-directed rehabilitation (EPDR) compared with standard (delayed) rehabilitation results in less shoulder pain and disability at 12 weeks postrandomisation, following arthroscopic repair of full-thickness tears of the rotator cuff. Detailed information about the study can be found in the published trial protocol^11^ and in brief below. Here, we describe in detail the protocol for the health economic components of RaCeR 2, which involves three phases of work linking short-term to long-term outcomes and estimating the associated resource use and costs for each trial arm.

### Objective

To report the methods used for the design and conduct of a cost consequence and cost-effectiveness analysis (CEA) comparing individualised EPDR (intervention) to standard (delayed) rehabilitation (control) after surgical repair of full-thickness tears of the rotator cuff of the shoulder from an NHS and personal social services perspective for England.

### RaCeR 2 trial summary

RaCeR 2 is a pragmatic, open-label, parallel-design RCT investigating two alternative approaches to rehabilitation following arthroscopic rotator cuff repair surgery. We aim to recruit 638 adults (18+ years) who have undergone arthroscopic surgical repair of a full-thickness tear, of any size, of their shoulder rotator cuff across 30 NHS orthopaedic and physiotherapy services in the UK, with the trial duration planned for 56 months beginning in September 2022.^11^

The intervention, EPDR, is an individualised approach where shoulder movement, sling removal and exercise are progressed as the participant feels able to and within their own levels of pain experience and tolerance, with support from a physiotherapist. The control consists of standard (delayed) rehabilitation, wherein the participant is advised to wear their sling for 4 weeks postsurgery, except for when eating, washing, dressing or undertaking the prescribed passive movement exercises.^11^

The primary outcome measure is shoulder pain and disability at 12 weeks postrandomisation, measured using the shoulder pain and disability index (SPADI) patient-reported outcome measure.^12^ Secondary outcome measures include shoulder pain and disability at 6 and 12 months postrandomisation; generic health-related quality of life (HRQoL) at 12 weeks, 6 and 12 months postrandomisation; time to return to usual activities at 12 weeks, 6 and 12 months postrandomisation (self-reported); healthcare resource use at 12 weeks, 6 and 12 months postrandomisation (self-reported); rotator cuff repair integrity at 12 months postrandomisation (diagnostic ultrasound scan); the number and nature of adverse events within 12 months postrandomisation (clinician and self-reported); and time spent out of the sling, measured in hours over 4 weeks postsurgery (self-reported). Please refer to the full published trial protocol^11^ for additional study details.

## Methods and analysis

The health economic analysis is made up of three phases:

1. Development of an initial conceptual cost-effectiveness model structure,
2. Within-trial cost consequences analysis (CCA), and
3. Long-term model-based CEA.

Below we describe the methods and analysis for each health economics component in detail. In line with the clinical effectiveness analysis, our base case analysis will be undertaken according to the intention-to-treat principle.^11^

### Phase 1: conceptual model structure

**Figure 1:**
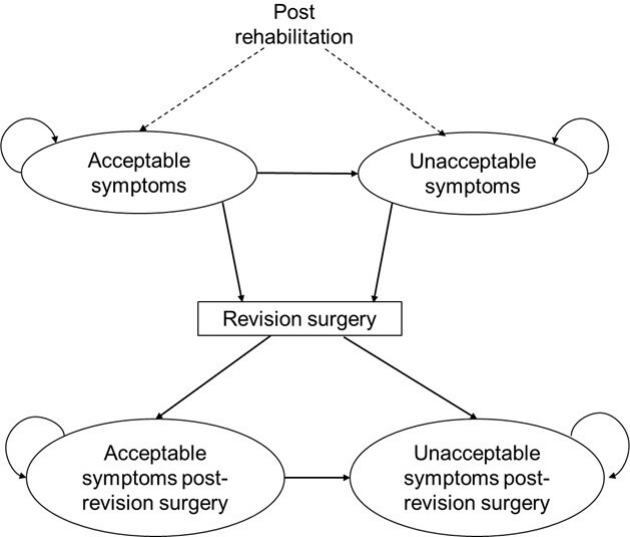
CEA conceptual model figure.

An initial conceptual cost-effectiveness model structure was developed to characterise the decision problem of the RaCeR 2 trial. This was informed by a targeted literature search conducted to identify published decision analytic models evaluating the cost-effectiveness of management strategies following surgery for the hip, knee or shoulder. Individual-level data from the original RaCeR pilot^5^ were analysed to determine the feasibility of estimating input parameters for the chosen model structure and to quantify the long-term costs and quality-adjusted life-years (QALYs) of individualised EPDR versus standard rehabilitation. However, given the limited sample size in the original RaCeR pilot,^5^ our results were inconclusive. After conducting a critical review of potentially relevant decision analytic models, a de novo model was developed to support the economic evaluation to be conducted as part of Phase 3 of the economic evaluation in RaCeR 2 (figure 1). This initial tentative structure differs from what has previously been used in the literature in two key ways: it reflects an additional ‘unacceptable symptoms’ health state and it incorporates patient-reported outcomes using the SPADI score, as described below. This model will be further refined once the RaCeR 2 trial data have been analysed.

The proposed Markov model structure includes two mutually exclusive health states that represent the prognosis of patients postrehabilitation. Following their primary rotator cuff repair surgery and rehabilitation period, patients will move into either an acceptable or unacceptable symptoms state. Those in the acceptable symptoms state may remain in that state until the model ends or may later move to the unacceptable symptoms state and/or require revision surgery for their rotator cuff. Those who move to the unacceptable symptoms state postrehabilitation may remain in that state until the model ends, or they require revision surgery. Those that require a revision surgery will move into either an acceptable or unacceptable symptoms state postrevision surgery. Those in the acceptable symptoms state pre- or postrevision surgery will either remain in this state or may later move into the unacceptable symptoms state and will remain in that state. Those who move to the unacceptable symptoms state postrevision surgery will remain in that state until the model ends. Throughout the model, patients will remain at risk of all-cause and revision surgery-related death. As patients transition through these health states, they will accrue costs and HRQoL, which will be summed over their sojourn time and contribute to the estimation of the total costs and QALYs for the strategies being compared. Time in the model will evolve at discrete time intervals (ie, cycles) of 6 months. The analysis will adopt a lifetime time horizon, which reflects the total period over which we expect costs and benefits to differ across the strategies being compared. We provide additional details to support the analysis of the model in section Phase 3 below.

#### Classifying patients into symptom states

There is presently a paucity of evidence on how to classify patients as having acceptable or unacceptable symptoms using clinically relevant patient-reported outcome measures such as their SPADI score. Measures of clinical relevance may include the minimally clinically important difference (MCID), patient acceptable symptoms state (PASS) or substantial clinical benefit.^13 14^ Various MCID thresholds have been reported for the SPADI, ranging from 8 to 14.^15 16^ Yet, it remains unclear whether MCID is the appropriate treatment target postsurgery, as there remains ambiguity in its interpretation and application.^17^ On the other hand, only a single study exists that assesses the presence/strength of longitudinal association between SPADI and PASS.^14^ Yet, this study also found low positive and negative predictive values, suggesting the reported thresholds may not be useful to use as a treatment target.^14^

Given these findings, there remains both structural and parameter uncertainty in the decision analytic model described in figure 1, with reference to our ability to classify postsurgical patients into clinically meaningful health states. We will therefore use patient-reported SPADI scores from the RaCeR 2 trial data to explore various thresholds of acceptable and unacceptable symptom states and test the uncertainty in our approach using scenario and value of information analyses, as described in the corresponding sections below.

### Phase 2: within-trial cost consequence analysis

The within-trial CCA will be conducted by analysing the individual-level data collected alongside RaCeR 2 to estimate the accrued resource use, costs and resulting health outcomes (relevant to the economic analysis) for each arm of the trial. CCA is a form of economic evaluation where costs and outcomes are presented in a disaggregated and transparent format.^18^ This enables decision-makers and other key stakeholders to note the relevance and relative importance of costs and outcomes as they relate to their own context.^18^ A descriptive table will be used to present mean health benefits, healthcare resource use and cost estimates, with appropriate measures of dispersion, for the intervention and control arm in the trial. Health benefits will be quantified in terms of HRQoL, measured by the EuroQoL 5-Dimension, 5-Level (EQ-5D-5L). The time horizon of the within-trial analysis is 12 months postrandomisation; costs and outcomes will therefore not be discounted.^19^

#### Health benefits

The key measure of health benefits for the economic analyses in RaCeR 2 is HRQoL using the EuroQoL 5-Dimension, 5-Level (EQ-5D-5L).^20^ The EQ-5D-5L is a generic preference-based self-reported outcome measure that describes HRQoL across five domains: mobility, self-care, usual activities, pain or discomfort, and anxiety or depression, each with severity levels ranging from 1 to 5, representing: no problems, slight problems, moderate problems, severe problems or unable to do.^20^ The EQ-5D is the preferred measure for HRQoL in the UK; however, the National Institute for Health and Care Excellence (NICE) does not presently recommend using the EQ-5D-5L value set for England, but rather the EQ-5D-3L (3-level) value set, to derive the *health state utility* values needed to support the analytical requirements for health economic studies.^19^ Therefore, the EQ-5D-5L will be mapped to 3 L using the mapping function developed by Hernández Alava *et al*,^21^ as recommended by NICE.^19^ Health state utility data represent preference-based measures of HRQoL. Values lie on a cardinal scale, where higher values (to a maximum of 1) represent a greater preference for the health state.^22^ Those with a value of 1 are considered in full health, while dead has a value of 0. Negative EQ-5D values represent health states considered worse than death.^22 23^

For Phase 2 of this study, descriptive health state values (eg, mean, standard deviation (SD), 95% confidence intervals (CI), minimum and maximum values) will be reported at baseline and follow-up points, by the trial arm.^24^

#### Resource use and costs

Healthcare resource use will be collected from participants during the RaCeR 2 trial using self-reported questionnaires at 12 weeks, 6- and 12 months postrandomisation. This data will inform the estimation of primary care services (eg, general practitioner, nurse and other NHS healthcare professionals), hospital services (eg, outpatient visits, emergency attendance and inpatient stay), diagnosis tests and prescribed drugs used for each patient in the study, by trial arm. The unit costs related to the reported resource utilisation will be estimated using the National Cost Collection for the NHS,^25^ the British National Formulary^26^ and the Unit Costs of Health and Social Care Manual.^27^ All costs will be estimated by multiplying the frequency of resource use by the cost per unit of use and will be reported using 2024 British Pounds. For Phase 2 of this study, the costs for each resource use category will be aggregated to obtain the total cost per patient over the trial period. Descriptive statistics (eg, mean, SD, 95% CI, minimum and maximum values) for costs will be reported by treatment arm. Indirect costs, including patient out-of-pocket expenditures for prescribed medications and time to return to usual activities (eg, work), will be reported separately as part of the CCA.

### Phase 3: long-term model-based cost-effectiveness analysis

Clinically relevant long-term events (eg, revision surgery) will be modelled to determine their impact on costs and health outcomes of individualised EPDR and standard (delayed) rehabilitation over a lifetime time horizon. Once data is available from the RaCeR 2 trial, the state-transition model structure developed in Phase 1 of this study will be updated as required, and parameters will be derived using both the trial data as well as published literature estimates, where relevant. Long-term costs and QALYs will be quantified by accruing the costs and HRQoL experienced by the patients over the model’s time horizon. Costs and outcomes incurred beyond the within-trial period (12 months postrandomisation) will be discounted at an annual rate of 3.5%, in line with NICE guidelines.^19^ Below, we detail the analysis plan that will be used to support Phase 3 of this study.

#### Health benefits

We will combine health state-specific utilities with the number of years lived in each health state to yield the QALYs representing the long-term benefits accrued within each arm of the trial. Note that QALYs are pertinent to Phase 3 of this study only, while Phase 2 of this study aims to report the costs and effects collected during the trial in a disaggregated and transparent format, a criterion of CCA.^18^

Health state utility data measured by the EQ-5D-3L are known to have several distinct characteristics, namely they are multimodal, bounded at 1, have a point probability mass at 1 and a substantial gap between one and the next value for those not in full health.^21 28^ These characteristics will be factored into statistical analysis, where a series of regression models designed to support these idiosyncrasies (eg, two-part models, beta models, adjusted limited dependent variable mixture models, generalised linear models (GLMs)) will be estimated and assessed using goodness-of-fit criteria.^28 29^ Regression models will include the following covariates: health state, age, sex, ethnicity, location of tear, size of tear, body mass index (BMI), highest level of education, employment status and SPADI score.

#### Resource use and costs

To estimate long-term resource use and costs, additional statistical analysis will take place using the trial data. Healthcare costs are often characterised by an asymmetric, right-skewed non-negative distribution, with occasionally many patients reporting zero costs,^30^ and there is no dominant method for analysis.^31^ We will therefore run several potential models from the GLM family and, using a link test and modified Parks test, identify the correct link function and distribution.^30^ Where necessary, we will additionally use two-part model extensions of the GLM for the analysis of cost data. All models will be adjusted for the following covariates: health state, age, sex, ethnicity, location of tear, size of tear, BMI, highest level of education, employment status and SPADI score. Using postestimate margins analysis, we will quantify the state-specific mean costs by treatment arm.^32^

### Missing data

Missing resource use, cost and HRQoL data is a common issue among health economic evaluations and may occur for various reasons.^24 33 34^ For our base case analysis, the mechanism of missingness will be assessed by examining whether study participants providing complete and incomplete observations differ systematically by sociodemographic (eg, age, sex, ethnicity, education, employment status) or symptomatic (eg, SPADI score, HRQoL score) characteristics. If missing data are associated with these characteristics, this would suggest that data are missing at random, and an appropriate imputation strategy will be used as recommended by Faria *et al*.^35^ In addition to our base case imputed analysis, we will also conduct a sensitivity analysis using a complete case analysis,^24 34^ the advantage of which is it enables one to view the results under imputed and non-imputed conditions, providing a greater understanding of the differences between, and impact of, observed and unobserved data on the overall analysis.^33^

### Subgroup analysis

Subgroup analysis will be conducted to explore heterogeneity in cost-effectiveness results by rotator cuff tear size.

### Sensitivity analysis

To address overall uncertainty in model parameters, a probabilistic sensitivity analysis (PSA) will be performed, where multiple model parameters, including probabilities, costs and utilities, will be varied simultaneously using suitable distributions (eg, beta, gamma, Dirichlet) of possible mean values as opposed to single point estimates.^23^ Using Monte Carlo simulations, we will quantify the joint distribution of the incremental costs and incremental QALYs of individualised EPDR versus standard (delayed) rehabilitation. Additional scenario analyses will take place to test additional model assumptions (eg, classification of patients into clinically meaningful symptom states, per protocol vs intention-to-treat analysis, complete case vs imputed analyses) and their impact on overall cost-effectiveness.

### Value of information analysis

A value of information (VOI) analysis will be conducted to characterise the potential risks or consequences associated with a decision to implement individualised EPDR rehabilitation within the NHS given the evidence produced by RaCeR 2, and to assess the value for money of conducting further research to reduce existing areas of uncertainty in decision-making.^36^ The VOI analysis will provide information on the expected value of perfect, partial perfect or sample information in relation to the additional cost of obtaining this information through additional research.^36^

### Patient and public involvement

Patient and public involvement (PPI) was an embedded component in the RaCeR pilot,^5^ and our PPI group continues to be actively involved in all stages of RaCeR 2, such as in supporting the classification of patients into symptom states for our Markov state transition model. We will also work collaboratively to cocreate dissemination materials that are tailored for members of the public. Our PPI lead (MM) holds regular engagement meetings with our PPI group.

## Ethics and dissemination

A favourable ethical review was granted by the London-Stanmore Research Ethics Committee (23/LO/0195) on 13 April 2023. Cost-effectiveness findings from RaCeR 2 will be shared with stakeholders via peer-reviewed publications and presentations at national and international conferences. The Consolidated Health Economic Evaluation Reporting Standards (CHEERS) 2022 checklist will be used to guide the reporting of final economic evaluation findings.^37^ The RaCeR 2 website (www.racer2study.co.uk) is our main hub providing access to videos of the exercises. Videos with the trial results will be made available on the website that can be used to inform patients, clinicians and policy decision-makers.^11^
