# Supplementary material for: Protocol for the economic evaluation of individualised (early) patient-directed rehabilitation versus standard rehabilitation after surgical repair of the rotator cuff of the shoulder (RaCeR 2)
Source: BMJ Open. 2025 Jun 20;15(6):e097469. doi: 10.1136/bmjopen-2024-097469 (PMC12182128; doi:10.1136/bmjopen-2024-097469)
Supplement: Supplementary file 1 [file bmjopen-2024-097469.reviewer_comments.pdf]

PEER REVIEW HISTORY

BMJ Open publishes all reviews undertaken for accepted manuscripts. Reviewers are asked to complete a checklist review form and are provided with free text boxes to elaborate on their assessment. These free text comments are reproduced below.

ARTICLE DETAILS

Title (Provisional)

Protocol for the economic evaluation of individualised (early) patient-directed rehabilitation versus standard rehabilitation after surgical repair of the rotator cuff of the shoulder (RaCeR2)

Authors

Manca, Andrea; Premji, Shainur; Gc, Vijay S.; Chen, T; Mazuquin, Bruno; Fakis, Apostolos; Bateman, Marcus; Moffatt, Maria; Littlewood, Chris

---

VERSION 1 - REVIEW

**Reviewer**	**1**
**Name**	**Paloneva, Juha**
**Affiliation**	**Central Finland Central Hospital, Department of Surgery**
**Date**	**02-Feb-2025**
**COI**	**None**

The authors report a protocol for the secondary analysis of a randomised controlled trial that compares two different post-operative rehabilitation methods for patients who have undergone rotator cuff repair surgery.

It is important to note that several high-quality RCTs comparing surgical and non-surgical treatment of full-thickness rotator cuff tears have been published in recent years. The evidence suggests that surgery may not be superior to non-surgical treatment for rotator cuff tendon tears, at least in cases of non-traumatic tears. If surgery itself is not demonstrably effective, what then is the significance of the post-operative rehabilitation method? The authors should address this issue and reference the relevant literature (Cederqvist et al., Moosmayer et al., Kukkonen et al., Lambers-Heerspink et al., Ranebo et al.).

Additionally, the authors should clarify whether both traumatic and non-traumatic tendon tears are included in their analysis, as the aetiology may influence the outcome.

It should be clearly stated that this is a secondary analysis of the RACER2 trial. Furthermore, the power calculations are based on the SPADI score rather than cost outcomes, which represents a limitation that should be clearly acknowledged.

If my understanding is correct, this trial assessed only direct costs. However, indirect costs—such as those associated with sick leave and loss of productivity—may significantly exceed direct costs for both the patient and society. The authors should clearly state whether indirect costs were not included in the analysis.

The cost of care is highly dependent on the social and healthcare system of a given country. As a result, an economic evaluation conducted in one setting may not be directly applicable to other countries or healthcare systems. This limitation should be acknowledged.

Minor Issues

Introduction, page 5: The authors state that “While surgical techniques have progressed, the optimal approach to rehabilitation following surgery to repair the torn rotator cuff remains unknown (5,9).” They cite a study by Funk L: “Arthroscopic shoulder surgery has progressed, has the rehabilitation? Int Musculoskelet Med. 2012 Dec;34(4):141–5.” However, this paper is relatively old and more of an opinion piece. Moreover, reference 5 does not contain data supporting this claim, nor do the papers it cites. I believe there have not been significant advancements in surgical techniques in recent years. The authors may wish to revise this statement and ensure the references are appropriate.

Page 11: The authors state that regression models will include ethnicity as a covariate. How is ethnicity measured or categorised in the study? Is its inclusion as a covariate justified?

Page 12: Regarding the subgroup analysis, the authors should clarify the definition of “rotator cuff tear size.” Does this refer to the number of affected tendons, the tear size in millimetres, or another parameter?

Reviewer **2**

Name **Wagner, Adam**

Affiliation **NIHR Applied Research Collaboration (ARC) East of England (EoE)**

Date **13-Mar-2025**

COI **None**

Thank you for the opportunity to read this well written, robust and interesting health economic analysis plan (HEAP). I have minor points/suggestions for the authors to consider.

Minor

P.5, 26-31: Detail on the intervention to be evaluated is very brief. I appreciate interested readers can go to other materials for further detail, but could some further detail be provided here to make the HEAP more self-contained? For instance, what does the “support from a physiotherapist” consist of? How many sessions? Weekly? Particular grade of physio? In person, or by virtual communication? Please consider adding a few further sentences, particularly that might inform with respect to resource implications.

P.6, L43-44: You comment: “Following a critical review of potentially relevant decision analytic models” – was this critical review published? If yes, please reference. Irrespective, please add a few sentences to summarise/highlight the deficiencies in these other models that required you to develop your own.

P.8, L20-39: Please add a few sentences to motivate/explain why a CCA is adopted here, rather than a cost-utility analysis (CUA). (I would not suggest that this more limited analysis should have precedence over the model that is likely to more comprehensively capture costs and benefits, but I am not sure it is immediately obvious why one could not do CUA for the within-trial analysis /and/ the model.

P.10, L26-31 and related to the above point: Why not report QALYs as part of the CCA? Please add a sentence to motivate. If you continue to exclude QALYs from the CCA, it may be useful to note/signpost this point in the earlier section (e.g. P.8, L20-39).

The Consolidated Health Economic Evaluation Reporting Standards (CHEERS) 2022 checklist is not referenced – is this a conscious choice? If yes, please justify. Otherwise, please consider whether this or some other checklist should be utilised.

Very minor:

p.5, L43-58: Separate elements of list of secondary outcomes with semi-colons.

p.5, L47-50, “time to return to usual activities at 12 weeks, 6 and 12 months post-randomisation (self-reported)” – the wording sounded a little odd/unclear to me – at each of these timepoints, is there a binary indicator to the effect of “Have you returned to usual activities? Y/N” – or does it ask about “At what time during this period did you return to usual activities?” Please consider whether some wording might clarify.

VERSION 1 - AUTHOR RESPONSE

It is important to note that several high-quality RCTs comparing surgical and non-surgical treatment of full-thickness rotator cuff tears have been published in recent years. The evidence suggests that surgery may not be superior to non-surgical treatment for rotator cuff tendon tears, at least in cases of non-traumatic tears. If surgery itself is not demonstrably effective, what then is the significance of the post-operative rehabilitation method? The authors should address this issue and reference the relevant literature

(Cederqvist et al., Moosmayer et al., Kukkonen et al., Lambers-Heerspink et al., Ranebo et al.).

We recognise the published RCTs and systematic reviews that report no difference in clinical outcomes between surgical repair of the rotator cuff and non-surgical treatment for some patients. However, in the UK alone over 6,500 surgical repairs of the rotator cuff are performed annually, and this number continues to rise. So, despite this research evidence, it is apparent that some patients are still undergoing surgery and there is uncertainty relating to the most clinically and cost-effective approach to post-surgical rehabilitation. This is the research question we have been funded to answer. The issue of the effectiveness of surgical repair of the rotator cuff versus non-surgical treatment is a different research question which we are not aiming to answer through our RaCeR 2 study. Thus, we regard this as a moot point and have opted not to amend the manuscript.

Additionally, the authors should clarify whether both traumatic and non-traumatic tendon tears are included in their analysis, as the aetiology may influence the outcome.

As the reviewer recognises, a traumatic or non-traumatic onset might be one of many factors that influence clinical outcome, although this has been questioned recently (https://pmc.ncbi.nlm.nih.gov/articles/PMC10838469/). Our eligibility criteria are purposefully pragmatic and, as stated in the manuscript, we will include adults (18+ years) awaiting arthroscopic surgical repair of a full-thickness tear, of any size, of their shoulder rotator cuff. Hence, patients with both a traumatic and non-traumatic onset are eligible to be included and because the mechanism of onset is one of many potential factors that might influence outcome, we have opted not to add further detail to maintain alignment with our published RCT protocol (https://bmjopen.bmj.com/content/14/4/e081284).

It should be clearly stated that this is a secondary analysis of the RACER2 trial.

Thank you for your feedback. We report the protocol for the primary analysis of the health economics data in the RaCeR-2 trial. This is not a secondary analysis, despite costs and HRQoL being secondary outcomes, as it is usually the case in economic evaluation conducted alongside RCTs. We have opted not to amend the manuscript, accordingly.

Furthermore, the power calculations are based on the SPADI score rather than cost outcomes, which represents a limitation that should be clearly acknowledged.

We do not consider this to be a limitation of this study, as it is common for cost-effectiveness analyses to be carried out on RCTs whose power calculations are based on the statistical significance of the primary clinical outcome. The common view in the health economics community is that rules of inference are arbitrary, as policy decisions centre around resource

use and costs, in addition to clinical outcomes (https://pubmed.ncbi.nlm.nih.gov/10537899/). In other words, despite not conducting power calculations for resource use and cost outcomes, there will still be costs incurred by the health system irrespective of which treatment arm is ultimately adopted; our analysis attempts to measure these costs and frame them within the context of an overall budget constraint, to inform policy decisions. We have opted not to amend the manuscript according to this feedback.

If my understanding is correct, this trial assessed only direct costs. However, indirect costs—such as those associated with sick leave and loss of productivity—may significantly exceed direct costs for both the patient and society. The authors should clearly state whether indirect costs were not included in the analysis.

As this study will be undertaken using an NHS and personal social services perspective, productivity and sick leave costs will not be included as part of the long-term cost-effectiveness analysis. However, indirect costs (e.g. patient out of pocket health costs) are being collected as part of this study and will be reported as part of the CCA. We have added this detail to the resource use and costs section for Phase 2 of the study.

The cost of care is highly dependent on the social and healthcare system of a given country. As a result, an economic evaluation conducted in one setting may not be directly applicable to other countries or healthcare systems. This limitation should be acknowledged.

We agree that an economic evaluation conducted in one setting may not be directly applicable to other settings, however, in this case we are conducting the economic evaluation from the perspective of our decision maker, which is the NHS for England and Wales. To facilitate considerations about the generalisability of our results to other settings, we will also report our results in a disaggregated manner (CCA) prior to using a decision analytic model to estimate the long-term costs and consequences (CEA). We have opted not to include this as a limitation in our manuscript, accordingly.

Introduction, page 5: The authors state that "While surgical techniques have progressed, the optimal approach to rehabilitation following surgery to repair the torn rotator cuff remains unknown (5,9)." They cite a study by Funk L: "Arthroscopic shoulder surgery has progressed, has the rehabilitation? Int Musculoskelet Med. 2012 Dec;34(4):141–5." However, this paper is relatively old and more of an opinion piece. Moreover, reference 5 does not contain data supporting this claim, nor do the papers it cites. I believe there have not been significant advancements in surgical techniques in recent years. The authors may wish to revise this statement and ensure the references are appropriate.

In the UK, surgical techniques have advanced in recent years with a move from majority open to majority arthroscopic repair, hence we are comfortable with this statement. Also, reference 5, the report of our RaCeR pilot randomised controlled trial, and reference 10, cited in the same paragraph, which is our updated survey of current practice, reflect both the uncertainty and lack of change in rehabilitation so we have opted not to revise this.

Page 11: The authors state that regression models will include ethnicity as a covariate. How is ethnicity measured or categorised in the study? Is its inclusion as a covariate justified?

Thank you for your question. The questionnaire categorises ethnicity in accordance with what is reported by the Office for National Statistics in the UK: White - British, White - Irish, White - Gypsy, White - Roma, White - Other, Asian - Indian, Asian - Pakistani, Asian - Bangladeshi, Asian - Chinese, Asian - Other, Black - African, Black - Caribbean, Black - Other, Mixed - White and Black African, Mixed - White and Black Caribbean, Mixed - White and Asian, Mixed - Other, Arab, Other (open ended text).

The question whether inclusion of ethnicity as a covariate is justified is an important one. We have designed RaCeR 2 to be an inclusive study, and we are aiming to recruit a diverse sample. So, this is an opportunity to explore the influence of ethnicity given the current uncertainty. Hence, we accept the point but think that inclusion is justified from this perspective of uncertainty.

Page 12: Regarding the subgroup analysis, the authors should clarify the definition of "rotator cuff tear size." Does this refer to the number of affected tendons, the tear size in millimetres, or another parameter?

In our trial protocol, we have defined rotator cuff tear size according to convention, as follows:
- Small (<1cm),
- Medium (1cm to 3cm),
- Large/massive (>3cm),
- Unknown

P.5, 26-31: Detail on the intervention to be evaluated is very brief. I appreciate interested readers can go to other materials for further detail, but could some further detail be provided here to make the HEAP more self-contained? For instance, what does the "support from a physiotherapist" consist of? How many sessions? Weekly? Particular grade of physio? In person, or by virtual communication? Please consider adding a few further sentences, particularly that might inform with respect to resource implications.

In line with feedback provided by the Editor, we have reduced the level of detail surrounding the trial protocol and instead refer readers to this protocol for further details.

P.6, L43-44: You comment: "Following a critical review of potentially relevant decision analytic models" – was this critical review published? If yes, please reference. Irrespective, please add a few sentences to summarise/highlight the deficiencies in these other models that required you to develop your own.

This review was not published, but rather internally discussed among a team of clinicians and health economists. Other decision analytic models (e.g. https://pubmed.ncbi.nlm.nih.gov/9885464/) have highlighted health states based on the success of primary surgery. Our model differs in two key ways: we have added an additional 'unacceptable symptoms' health state to the model, and we have opted to use patient reported outcome measures (the SPADI) to define health states. We have added additional detail to the Phase 1 model overview text to reflect this.

P.8, L20-39: Please add a few sentences to motivate/explain why a CCA is adopted here, rather than a cost-utility analysis (CUA). (I would not suggest that this more limited analysis should have precedence over the model that is likely to more comprehensively capture costs and benefits, but I am not sure it is immediately obvious why one could not do CUA for the within-trial analysis /and/ the model.

As we noted within the CCA section, "CCA is a form of economic evaluation where costs and outcomes are presented in a disaggregated and transparent format (19). This enables decision-makers and other key stakeholders to note the relevance and relative importance of costs and outcomes as it relates to their own context (19)". In other words, if one were to conduct a CUA for the within trial analysis, the costs and outcomes would not be reported in a disaggregated format, but rather they would be modelled alongside other forms of evidence relevant to the overall policy context. The benefit of completing a CCA, followed by a CEA/ CUA for the long-term analysis, is that we are first reporting RCT-specific costs and outcomes, and then placing this RCT evidence within a broader framework of evidence synthesis and decision analysis, designed to support key policy decisions within an overall healthcare context (https://pubmed.ncbi.nlm.nih.gov/16491461/).

P.10, L26-31 and related to the above point: Why not report QALYs as part of the CCA? Please add a sentence to motivate. If you continue to exclude QALYs from the CCA, it may be useful to note/signpost this point in the earlier section (e.g. P.8, L20-39).

As above, QALYs at 12 months assume everyone dies by the end of follow up. Which is clearly not the case. Therefore, calculating QALYs for within trial economic evaluation is

misleading, which is why we are not conducting a CUA/CEA for the within trial period. We have opted not to make this amendment to the manuscript.

The Consolidated Health Economic Evaluation Reporting Standards (CHEERS) 2022 checklist is not referenced – is this a conscious choice? If yes, please justify. Otherwise, please consider whether this or some other checklist should be utilised.

The CHEERS 2022 checklist was design for the reporting of economic evaluation results. We have therefore not used this checklist for the reporting of this protocol; we can, however, confirm that all items listed in CHEERS for the Abstract, Title, and Methods sections have been reported as part of this protocol, and that CHEERS will be used to guide the reporting of the final economic evaluation findings.

p.5, L43-58: Separate elements of list of secondary outcomes with semi-colons.

Thank you for your feedback. We have made this change as suggested.

p.5, L47-50, "time to return to usual activities at 12 weeks, 6 and 12 months post-randomisation (self-reported)" – the wording sounded a little odd/unclear to me – at each of these timepoints, is there a binary indicator to the effect of "Have you returned to usual activities? Y/N" – or does it ask about "At what time during this period did you return to usual activities?" Please consider whether some wording might clarify.

The wording does differ depending on the question, which is why we have reported this consistently with what is reported in the trial protocol. For example, participants can indicate yes/no for whether they have returned to driving, but when asked whether they have had to take time off from their usual activities due to their injury (yes/no), this is then followed up with a question asking how many days they had to take off.

VERSION 2 - REVIEW

**Reviewer**	**2**
**Name**	**Wagner, Adam**
**Affiliation**	**NIHR Applied Research Collaboration (ARC) East of England (EoE)**
**Date**	**20-Apr-2025**
**COI**	

Thank you for the opportunity to review this re-submission.

I believe the authors have responded well to our feedback and do not think further amendments are necessary.

I would just briefly note that my suggestion on using the CHEERS checklist was in relating to noting its use in the proposed work, /not/ applying it to this article/protocol. The authors helpfully note their intention to use CHEERS in the proposed work in their response – they might consider noting this in the article.

VERSION 2 - AUTHOR RESPONSE

Reviewer 2:
I would just briefly note that my suggestion on using the CHEERS checklist was in relating to noting its use in the proposed work, /not/ applying it to this article/protocol. The authors helpfully note their intention to use CHEERS in the proposed work in their response – they might consider noting this in the article.

We have made the recommended change to the "Ethics and dissemination" section of the manuscript.
